# Personal carbon monoxide exposure, respiratory symptoms, and the potentially modifying roles of sex and HIV infection in rural Uganda: a cohort study

**DOI:** 10.1186/s12940-019-0517-z

**Published:** 2019-08-20

**Authors:** Crystal M. North, Piers MacNaughton, Peggy S. Lai, Jose Vallarino, Samson Okello, Bernard Kakuhikire, Alexander C. Tsai, Marcia C. Castro, Mark J. Siedner, Joseph G. Allen, David C. Christiani

**Affiliations:** 1 Division of Pulmonary and Critical Care Medicine, Massachusetts General Hospital, 55 Fruit Street, BUL-148, Boston, MA 02118 USA; 2 Harvard T.H. Chan School of Public Health, Boston, MA USA; 3 Harvard Medical School, Boston, MA USA; 4 Mbarara University of Science and Technology, Mbarara, Uganda; 5 University of Virginia Health System, Charlottesville, USA

**Keywords:** Africa, Biomass, Pulmonary, Air pollution, Global health

## Abstract

**Background:**

Most of the global burden of pollution-related morbidity and mortality is believed to occur in resource-limited settings, where HIV serostatus and sex may influence the relationship between air pollution exposure and respiratory morbidity. The lack of air quality monitoring networks in these settings limits progress in measuring global disparities in pollution-related health. Personal carbon monoxide monitoring may identify sub-populations at heightened risk for air pollution-associated respiratory morbidity in regions of the world where the financial cost of air quality monitoring networks is prohibitive.

**Methods:**

From September 2015 through May 2017, we measured 48-h ambulatory carbon monoxide (CO) exposure in a longitudinal cohort of HIV-infected and uninfected adults in rural southwestern Uganda. We fit generalized mixed effects models to identify correlates of CO exposure exceeding international air quality thresholds, quantify the relationship between CO exposure and respiratory symptoms, and explore potential effect modification by sex and HIV serostatus.

**Results:**

Two hundred and sixty study participants completed 419 sampling periods. Personal CO exposure exceeded international thresholds for 50 (19%) participants. In covariate-adjusted models, living in a home where charcoal was the main cooking fuel was associated with CO exposure exceeding international thresholds (adjusted odds ratio [AOR] 11.3, 95% confidence interval [95%CI] 4.7–27.4). In sex-stratified models, higher CO exposure was associated with increased odds of respiratory symptoms among women (AOR 3.3, 95%CI 1.1–10.0) but not men (AOR 1.3, 95%CI 0.4–4.4). In HIV-stratified models, higher CO exposure was associated with increased odds of respiratory symptoms among HIV-infected (AOR 2.5, 95%CI 1.01–6.0) but not HIV-uninfected (AOR 1.4, 95%CI 0.1–14.4) participants.

**Conclusions:**

In a cohort in rural Uganda, personal CO exposure frequently exceeded international thresholds, correlated with biomass exposure, and was associated with respiratory symptoms among women and people living with HIV. Our results provide support for the use of ambulatory CO monitoring as a low-cost, feasible method to identify subgroups with heightened vulnerability to pollution-related respiratory morbidity in resource-limited settings and identify subgroups that may have increased susceptibility to pollution-associated respiratory morbidity.

**Electronic supplementary material:**

The online version of this article (10.1186/s12940-019-0517-z) contains supplementary material, which is available to authorized users.

## Background

Air pollution, the leading environmental cause of morbidity and mortality worldwide, is responsible for over 7 million deaths annually [1, 2]. Cooking-related biomass exposure alone causes almost 4 million deaths each year [2]. Most of the global burden of pollution-related morbidity and mortality is believed to occur in resource-limited settings (RLS) [3], where ground-level air quality estimates are sparse due to limitations in the human capital and infrastructure necessary to establish and maintain air monitoring networks. This has limited scientific advancement in identifying vulnerable populations and quantifying the burden of pollution-related health impacts among the most affected portion of the global population.

Most of the 1 billion people with chronic respiratory disease globally live in RLS such as sub-Saharan Africa [4], where the convergence of the air pollution and HIV epidemics may underlie the disproportionate regional burden of disease. Although people living with HIV (PLWH) are more likely to have chronic lung disease due to virally-mediated lung damage and higher susceptibility to lung infections [5, 6], air pollution may synergistically increase lung disease risk in co-exposed populations through shared inflammatory pathways [7] and/or increased tuberculosis risk [8]. Recent data also suggest that the higher burden of air pollution-related respiratory symptoms among women in RLS – often attributed to higher exposure among women due to cooking-related biomass burning – may at least partially result from sex hormone-based differences in the pulmonary effects of inhaled pollutants [9, 10]. Despite these plausible relationships, little is known about whether these potentially vulnerable populations are at heightened risk for pollution-associated respiratory morbidity.

The scant air quality data in sub-Saharan Africa are almost exclusively from stationary samplers in urban or peri-urban centers [11], yet most of the African population lives in rural settings [12]. Furthermore, personal exposure to air pollution may not be accurately reflected by outdoor or household stationary air monitoring sites [13], which may introduce misclassification bias into studies of pollution-related health risks. Thus, while the Global Burden of Disease investigators have recently completed the first global estimates of ambient air pollution [1], estimates across sub-Saharan Africa may not reflect individual-level air pollution exposure. It is important to address this gap in knowledge because, until we have, we are ill equipped to identify and resolve global disparities in pollution-associated health effects.

Ambulatory carbon monoxide monitoring is an inexpensive, technically feasible method by which to quantify personal air pollution exposure in sub-Saharan Africa, where ground-level monitoring networks are absent. Carbon monoxide (CO) is a byproduct of the partial combustion of carbon-containing materials, and the major sources of CO exposure in RLS include biomass burning and traffic-related emissions [14–17]. In some studies, ambulatory CO exposure in RLS has been shown to correlate with particulate matter concentrations [17–20]. Chronic CO exposure is associated with respiratory symptoms, lung disease exacerbations, and mortality [21–23]. To test our hypothesis, we assessed the feasibility of using ambulatory CO monitoring to measure personal air pollution exposure in rural southwestern Uganda, identified correlates of higher CO exposure, and explored whether relationships between CO exposure and chronic respiratory symptoms differed based on sex or HIV serostatus.

## Methods

The Uganda Non-Communicable Diseases and Aging Cohort (UGANDAC) is a prospective, longitudinal observational cohort study of HIV infected and uninfected adults based in Mbarara District, Uganda [24–26]. Mbarara district, located in southwestern Uganda, includes an urban center and a larger surrounding rural area totaling 1846 km^2^. Most residents live in outlying rural areas, have at most a primary school education, and the local economy is dominated by petty trading, subsistence agriculture, and animal husbandry [27]. Food and water insecurity are common [28, 29]. In 2018, Uganda ranked 181st out of 192 for Gross National Income per capita, similar to most other sub-Saharan African countries [30].

The UGANDAC cohort is a mixed cohort of HIV-infected and uninfected adults. HIV-infected participants are at least 40 years of age, have been on antiretroviral therapy for at least 3 years, and receive their HIV-related care at the Mbarara Regional Referral Hospital’s HIV clinic. The HIV clinic is a prototypical, PEPFAR-supported, government-run HIV clinic. Sex and age-similar (by quartile of the HIV infected sub-group) HIV-uninfected participants were selected at random from a complete population census of a village located within the clinical catchment area and confirmed to be HIV uninfected prior to each annual study visit. There were no other inclusion or exclusion criteria. We collected information on demographics, medical comorbidities, socioeconomic status (asset ownership index) [31], smoking history [32], cooking fuel use, and respiratory symptoms [33] at yearly study visits (Additional file 1). We defined self-reported respiratory symptoms as chronic cough (a cough on most days for at least 3 months), dyspnea on exertion (difficulty breathing when hurrying on a flat ground or walking up a slight hill), or wheezing (ever having a wheezy or whistling sound in the chest). All questionnaires were administered in Runyankole, the locally spoken dialect, by trained study staff fluent in both English and Runyakole.

From September 2015 to October 2017, we measured 48 h of personal exposure to carbon monoxide (CO) at annual study visits using ambulatory CO monitors (EL-USB-CO Data Logger, Lascar Electronics Inc., Erie, PA). Monitors are lightweight (less than one ounce, 1-in. × 1-in. × 5-in.), cost less than $100 each, measure CO concentrations up to 1000 ppm with an internal resolution of 0.5 ppm, and are set to log CO concentrations every 60 s (www.dataq.com). Participants wore the CO monitor in a custom-made pouch at chest height for 48 h, beginning at the completion of study procedures at each annual study visit. Participants were instructed to place the CO monitors at their bedside during sleep and nearby during bathing-related activities. Data were stored electronically in the monitor and uploaded to a secured, password-protected database at the completion of each sampling period. A convenience sample of software-generated graphs of CO concentration over time were reviewed to confirm concentration variation over the sampling period as a surrogate for study procedure compliance.

Study procedures were approved by the Mbarara University of Science and Technology and Partners Healthcare human studies ethics committees. Consistent with national guidelines, we also received clearance for the study from the Uganda National Council for Science and Technology. All participants provided written informed consent.

### Statistical analysis

We first summarized cohort characteristics by HIV serostatus using Wilcoxon rank sum, chi-squared and Fisher’s exact tests, as appropriate. We evaluated for selection bias by comparing cohort characteristics between those who did versus those who did not complete at least one CO sampling session during the study period using Wilcoxon rank sum, chi-squared or Fisher’s exact tests, as appropriate.

We used the World Health Organization (WHO) air quality guidelines to define CO exposure thresholds. WHO guidelines stipulate that time-averaged CO exposure should not exceed 35 ppm over 1 h (1-h time-weighted average [TWA]) or 9 ppm over 8 h (8-h TWA) [34]. In studies of personal CO exposure, 1-h TWA has been shown to correlate with shorter-duration exposures while 8-h TWA has been shown to correlate with longer-duration exposures [35]. Thus, we classified participants as having CO exposure that exceeded WHO air quality thresholds if their highest CO exposure during the sampling period exceeded the 1-h TWA threshold of 35 ppm. We elected to use 1-h TWA to define exposure in our cohort because the most likely sources of CO in the study setting - traffic-related exposures while traveling on foot or by car and biomass exposures related to cooking, heating and lighting [36] activities - are generally shorter-term exposures. We identified the maximum 1-h TWA CO exposure in each sampling period by first calculating 1-h rolling averages across the entire 48-h sampling period and then identifying the maximum 1-h rolling average within the sampling period. To facilitate planned sensitivity analyses, we also calculated the maximum 8-h TWA CO exposure in each sampling period by first calculating 8-h rolling averages across the entire 48-h sampling period and then identifying the maximum 8-h rolling average within the sampling period. We evaluated the correlation between maximum 1-h and 8-h TWA CO concentrations using the Spearman correlation coefficient.

To identify correlates of personal CO exposure that exceeded WHO air quality thresholds, we fitted generalized mixed effects models, with a random effect intercept to account for repeated measures of CO within the same participant, in the following manner. First, we identified potential correlates of interest for CO exposure based on scientifically plausible relationships between the candidate covariate and the exposure of interest. These included environmental exposures such as biomass use (cooking fuel type, cooking distance from main home, home lighting type), season (dry versus rainy), home location (urban versus rural), smoking (current versus non-current), occupation, burning trash near the home, and home ventilation (number of windows in the home). We then evaluated relationships between each candidate covariate and the outcome of interest in unadjusted analyses; all covariates that had a *p-*value < 0.2 were then included in the final multivariable regression model. Participants with less than 8 h of CO data were excluded from these analyses. We evaluated the robustness of our results in a sensitivity analysis in which we defined CO exposure above WHO thresholds using the 8-h TWA rather than the 1-h TWA.

To evaluate the relationship between personal CO exposure and respiratory symptoms, we fitted generalized mixed effects models, with a random effect intercept to account for repeated measures of CO within the same participant, in the same manner as described above. We defined respiratory symptoms as self-reported chronic cough, dyspnea on exertion, or wheezing. In this model, 1-h TWA CO exposure above WHO thresholds was the primary explanatory variable of interest. We identified potential correlates of interest based on the scientific plausibility of their relationship with either the outcome (respiratory symptoms) or primary explanatory variable of interest (CO exposure). These included age, sex, smoking status, socioeconomic status (household asset index) [31], HIV serostatus, and season (dry versus rainy). We then used the same multivariable logistic regression model building technique as described above whereby we included all candidate covariates with a *p*-value < 0.2 in the final multivariable regression model, with the exception that we forced smoking status into the final model based on established relationships between smoking and respiratory morbidity.

We then evaluated for evidence of interaction between CO exposure and sex because of anticipated differences in CO exposure concentrations [20] and for evidence of interaction between CO exposure and HIV serostatus because of anticipated differences in respiratory symptom burden [37–39]. Beginning with the adjusted multivariable logistic regression model established through the above-described model building approach, we first conducted formal tests of interaction whereby we added CO*sex and CO*HIV interaction terms, independently, to the final model and evaluated their statistical significance. We then stratified models by HIV serostatus and by sex, respectively, to look for differential relationships between CO exposure and respiratory symptoms within strata.

Finally, we evaluated the robustness of our results in sensitivity analyses whereby we defined CO exposure above WHO thresholds using the 8-h TWA rather than the 1-h TWA. To evaluate whether CO exposure was simply a surrogate for biomass fuel type, we conducted additional sensitivity analyses in which 1) the CO exposure variable was replaced with the biomass variable, and 2) models were restricted to only those who lived in homes where firewood was the main cooking fuel rather than charcoal (biomass use for cooking is ubiquitous in the region) [40, 41]. Data were analyzed using R version 3.4.2 (R Project for Statistical Computing, Vienna, Austria).

## Results

**Fig. 1:**
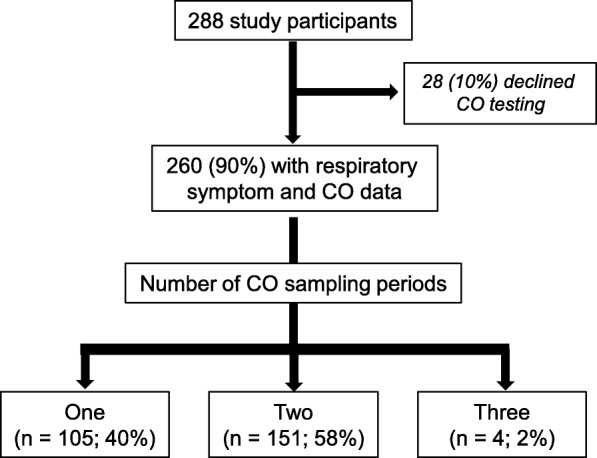
Flow diagram of study participant selection

A total of 419 sampling periods were completed among 260 (90%) of the 288 UGANDAC study participants (Fig. 1). Among the 260 study participants with at least one CO sampling period, 123 (47%) were women, 131 (50%) were HIV-infected, most (*n* = 221, 85%) lived in rural settings, and few (*n* = 41, 16%) were currently smoking (Table 1). Most of the cohort were subsistence farmers (*n* = 177, 68%) and had at most a primary school education (*n* = 234, 90%). Among the 131 participants with HIV, 122 (94%) had undetectable HIV viral loads, 105 (80%) had CD4 T-cell counts of at least 350 cells/mm^3^, and all had been taking antiretroviral therapy for a median of 9 years (interquartile range [IQR] 8–10). Biomass exposure was ubiquitous; 221 (85%) participants lived in homes where firewood was the main cooking fuel and 37 (14%) lived in homes that used mainly charcoal for cooking. The cohort was nearly evenly split between those who used primarily kerosene to light their homes (*n* = 141, 54%) and those who used primarily electricity, solar or battery-operated lights (*n* = 110, 42%). The above demographics are similar to the general Ugandan population with the exception of HIV prevalence, which is 5.9% nationally [42, 43]. Trash burning at home was reported by 162 (63%) of the cohort, most of whom did so less than once per week (*n* = 108, 62%) at a median distance from home of 20 m (IQR 10 - 50). The 28 participants (10%) who declined CO measurement had higher educational attainment than those who had at least one CO measurement (*p* = 0.04), otherwise there were no substantive differences between study participants who declined CO measurement as compared to those with at least one CO sampling period (Additional file 1: Table S1).

**Table 1:** Cohort characteristics at baseline

	Total Cohort (*n* = 260)	HIV + (*n* = 131)	HIV – (*n* = 129)
Age, years	51 [48, 56]	52 [49, 56]	51 [48, 55]
Female sex	123 (47)	61 (47)	62 (48)
Smoking History			
Never Smoker	133 (51)	73 (56)	60 (47)
Former Smoker	86 (33)	47 (36)	39 (30)
Current Smoker	41 (16)	11 (8)	20 (23)
Years of Smoking^a^	20 [10, 32]	16 [10, 25]	26 [11, 35]
Farmer	177 (68)	70 (53)	107 (83)
Rural dwelling	221 (85)	92 (70)	129 (100)
Education			
Did not complete primary school	143 (55)	63 (48)	80 (62)
Completed primary school	91 (35)	53 (40)	38 (29)
Completed secondary school	26 (10)	15 (11)	11 (9)
HIV Characteristics			
HIV viral load, copies/μL			
Undetectable		122 (94)	
Detectable, up to 10,000		6 (5)	
> 10,000		2 (2)	
CD4 T-cell count, cells/mm^3^			
≥ 500		54 (41)	
350–499		51 (39)	
< 350		26 (20)	
Taking antiretroviral therapy		131 (100)	
Antiretroviral therapy duration, years		9 [8, 10]	
Self-reported personal air quality			
Excellent or Very good	143 (55)	75 (57)	68 (53)
Fair	69 (27)	36 (27)	33 (26)
Poor or Very poor	48 (18)	20 (15)	28 (22)
Cooking fuel			
Firewood	221 (85)	94 (73)	127 (98)
Charcoal	37 (14)	35 (27)	2 (2)
Cooking location			
Inside the main house	6 (2)	6 (5)	0 (0)
Inside a separate structure	232 (89)	107 (82)	125 (97)
Outside	22 (8)	18 (14)	4 (3)
Distance from house, meters^b^	1 [0, 5]	1 [0, 5]	4 [3, 5]
Trash burning near home			
None	98 (38)	55 (42)	43 (33)
< 1 times/week	108 (42)	47 (36)	61 (47)
1–6 times/week	52 (20)	29 (22)	23 (18)
At least daily	2 (1)	0 (0)	2 (2)
Distance from home, meters^c^	20 [10, 50]	20 [10, 50]	20 [10, 50]
Home lighting source			
Electricity, solar power	84 (32)	62 (47)	22 (17)
Kerosene	141 (54)	41 (31)	100 (78)
Battery	26 (10)	20 (15)	6 (5)
Candles	6 (2)	5 (4)	1 (1)
Home ventilation			
Rooms in house	3 [3, 4]	3 [2, 4]	4 [3, 4]
Windows in house	4 [3, 5]	4 [2, 4]	4 [4, 5]
Doors in house	2 [2, 2]	2 [1, 2]	2 [2, 2]

Median [IQR], n (%) unless otherwise indicated

^a^ Current/former smokers only; ^b^ For those who don’t cook in the main house; ^c^ For those who burn trash near their homes

**Fig. 2:**
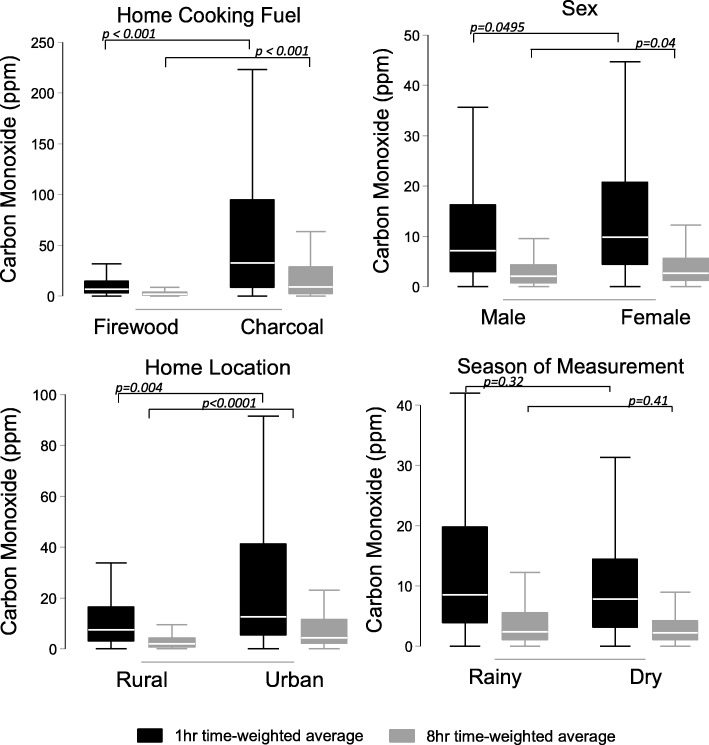
Box plots represent median, 25th and 75th percentiles of carbon monoxide exposure concentrations

Among the 260 study participants who completed CO sampling periods, 105 (40%) completed one sampling period, 151 (58%) completed two sampling periods, and four (2%) completed three sampling periods. Sampling periods were nearly evenly split between the dry and rainy seasons (184 [44%] vs. 235 [56%]). Median 24-h CO exposure was 1.5 ppm (IQR 0.4–3.1), median 1-h time-weighted average (TWA) CO exposure was 8.2 ppm (IQR 3.5–18.2), and median 8-h TWA CO exposure was 2.3 ppm (IQR 1.0–4.7). In unadjusted comparisons, ambulatory CO exposure was higher among participants living in homes that used charcoal for cooking as compared to firewood, among those who live in urban as compared to rural settings, among women as compared to men, and among people living with HIV (PLWH) as compared to HIV uninfected participants (Table 2, Fig. 2). Ambulatory CO levels exceeded WHO 1-h or 8-h guidelines at least once among 50 (19%) study participants in a total of 60 (14%) sampling periods. In multivariable-adjusted generalized mixed effects models, living in a home where charcoal as compared to firewood was the main cooking fuel was the only correlate of exposure to ambulatory CO concentrations exceeding WHO guidelines (adjusted odds ratio [AOR] 11.3, 95% confidence interval [95% CI] 4.7–27.4, *p* < 0.001) (Table 3). Effect estimates were similar when categorizing CO exposure above WHO guidelines using the 8-h rather than the 1-h TWA guideline (Additional file 1: Table S2).

**Table 2:** Mean CO exposure concentrations [IQR] during each sampling period, *n =* 419 total sampling periods

Characteristic	1-h TWA	*P* value^*^	8-h TWA	*P* value^*^
Sex		0.0495		0.04
Male (*n* = 220)	7.2 [2.9, 16.3]		2.1 [0.7, 4.4]	
Female (*n* = 199)	9.9 [4.4, 20.8]		2.7 [1.2, 5.6]	
Season		0.32		0.41
Dry (*n* = 184)	7.8 [3.2, 14.5]		2.2 [1.0, 4.3]	
Rainy (*n* = 235)	8.6 [3.8, 19.8]		2.4 [1.0, 5.5]	
Biomass		< 0.0001		< 0.0001
Charcoal (*n* = 60)	32.9 [9.0, 94.8]		9.2 [2.5, 28.9]	
Firewood (*n* = 354)	7.1 [3.1, 14.8]		2.1 [0.8, 4.2]	
Home location		0.0004		< 0.0001
Urban (*n* = 69)	12.7 [5.5, 41.3]		4.4 [2.2, 11.6]	
Rural (*n* = 350)	7.5 [3.2, 16.6]		2.1 [0.8, 4.3]	
HIV serostatus		0.0003		< 0.0001
HIV infected (*n* = 201)	10.4 [4.1, 27.3]		3.0 [1.4, 7.7]	
HIV uninfected (*n* = 218)	6.9 [3.1, 13.9]		1.9 [0.7, 3.7]	

*CO* Carbon monoxide, *IQR* Interquartile range, *TWA* Time-weighted average, *HIV* Human immunodeficiency virus

^*^Wilcoxon rank sum tests

**Table 3:** Correlates of 1-h time weighted average CO exposure > 35 ppm

Characteristic	Unadjusted		Adjusted	
	Odds Ratio	95% CI	Odds Ratio	95% CI
Biomass cooking - charcoal	15.4^***^	7.6, 31.5	11.3^***^	4.7, 27.4
Kerosene lighting	0.4^**^	0.2, 0.8	0.9	0.4, 2.0
Cooking distance from home^a^	1.0	0.9, 1.0		
Trash burning at home	0.6	0.3, 1.3	0.5	0.2, 1.1
Dry Season	0.8	0.4, 1.6		
Smoking	0.8	0.3, 1.9		
Urban residence	3.6^***^	1.8, 7.4	1.5	0.6, 3.7
Home ventilation^b^	0.7^***^	0.5, 0.8	0.8	0.7, 1.0
Farmer	0.3^**^	0.2, 0.7	0.9	0.4, 2.2

Reference categories: biomass - firewood; lighting – electricity, solar, or battery power; season - rainy; residence – rural

*CO* Carbon monoxide, *CI* Confidence interval, *HIV* Human immunodeficiency virus

^**^ *p* < 0.01; ^***^ *p* < 0.001

^a^per additional meter; ^b^ per additional window

Respiratory symptoms were common among the cohort. Self-reported chronic cough, dyspnea and/or wheezing were reported by 69 (27%) study participants. In models adjusted for sex, HIV serostatus, CO exposure and smoking status, the odds of self-reported respiratory symptoms were higher among women as compared to men (AOR 4.0, 95% CI 2.1 to 7.5, *p* < 0.001), and those with CO exposure over WHO guidelines had over twice the odds of any respiratory symptoms compared to those whose CO exposure did not exceed WHO guidelines (AOR 2.1, 95% CI 1.0–4.7, *p* = 0.06; Table 4). In models stratified by sex and HIV serostatus, CO exposure above WHO guidelines was associated with increased odds of respiratory symptoms among women (AOR 3.3, 95% CI 1.1–10.0, *p* = 0.03) but not among men and among PLWH (AOR 2.5, 95% CI 1.01–6.0, *p* = 0.048) but not HIV uninfected participants, although the corresponding interaction terms between CO exposure and either sex or HIV serostatus were not statistically significant (Tables 5 and 6). When considering each respiratory symptom individually, the relationship between higher CO exposure and respiratory symptoms was driven by dyspnea on exertion (AOR 3.4, 95% CI 1.3–9.0, *p* = 0.01), as no relationship was present with higher CO exposure and either cough or wheeze.

**Table 4:** Correlates of self-reported respiratory symptoms

Characteristic	Unadjusted		Adjusted	
	OR	95% CI	OR	95% CI
Age, per year	1.0	0.98, 1.05		
Female sex	2.7^***^	1.7, 4.2	4.0^***^	2.1, 7.5
HIV serostatus	1.7^*^	1.1, 2.7	1.1	0.6, 2.1
1-h CO > 35 ppm	2.0	0.9, 4.2	2.1^a^	1.0, 4.7
Dry season	0.8	0.5, 1.4		
Smoking Status				
Current	0.8	0.4, 1.7	1.1	0.4, 3.0
Former	1.2	0.8, 2.0	1.1	0.6, 2.0
Asset Index				
Poorest	1.1	0.6, 2.0		
Poorer	0.7	0.4, 1.3		
Richer	0.8	0.5, 1.5		

Reference categories: sex – male; HIV serostatus – HIV uninfected; season – rainy; asset index – richest; smoking status – never-smoker

*OR* Odds ratio, *CI* Confidence interval, *CO* Carbon monoxide, *HIV* Human immunodeficiency virus

^*^p < 0.05; ^***^ *p* < 0.001

^a^*p* = 0.06

**Table 5:** Correlates of self-reported respiratory symptoms, stratified by sex

Characteristic	Men *(220 sampling periods)*		Women *(200 sampling periods)*	
	Adjusted OR	95% CI	Adjusted OR	95% CI
HIV serostatus	4.7^*^	1.4, 16.3	0.6	0.3, 1.2
1-h CO > 35 ppm	1.3	0.4, 4.4	3.3^*^	1.1, 10.0
Smoking status				
Current	1.4	0.3, 6.2	2.8	0.4, 18.2
Former	1.4	0.5, 4.2	0.9	0.4, 1.9

Reference categories: sex - male; HIV serostatus - negative; smoking - never-smoker; biomass - firewood

*p* value for CO x sex interaction term *=* 0.70

*OR* Odds ratio, *CI* Confidence interval, *HIV* Human immunodeficiency virus, *CO* Carbon monoxide, *ppm* Parts per million

^*^p < 0.05

**Table 6:** Correlates of self-reported respiratory symptoms, stratified by HIV serostatus

Characteristic	HIV Positive *(202 sampling periods)*		HIV Negative *(218 sampling periods)*	
	Adjusted OR	95% CI	Adjusted OR	95% CI
Female sex	1.9	0.8, 4.3	11.2^***^	3.3, 38.0
1-h CO > 35 ppm	2.5^*^	1.0, 6.0	1.4	0.1, 14.4
Smoking status				
Current	2.6	0.6, 11.3	0.9	0.1, 5.0
Former	0.8	0.3, 2.0	1.3	0.6, 3.2

Reference categories: sex - male; HIV serostatus - negative; smoking - never-smoker; biomass – firewood

*p* value for CO x HIV interaction term = 0.42

*OR* Odds ratio, *CI* Confidence interval, *HIV* Human immunodeficiency virus, *CO* Carbon monoxide, *ppm* Parts per million

^*^p < 0.05

***p < 0.001

In sensitivity analyses, effect estimates were similar when using 8-h TWA limits to define WHO thresholds (Additional file 1: Tables S3-S5). Replacing the CO exposure variable with the biomass cooking variable halved the odds of respiratory symptoms associated with home cooking fuel type (AOR decreased from 2.1 to 1.0) but had no effect on other model covariates. Restricting the sample size to only those living in homes using firewood for cooking had no effect on model effect estimates (Additional file 1: Tables S6 and S7).

## Discussion

In a mixed cohort of people with and without HIV in rural southwestern Uganda, we found that personal CO exposure exceeded WHO air quality thresholds for one in five participants, that the odds of CO exposure exceeding air quality thresholds were over 10 times higher among those living in homes where charcoal was used for cooking, and that CO exposure was associated with respiratory symptoms among vulnerable populations such as women and those living with HIV.

Our study is among the first to compare personal CO exposure to WHO air quality thresholds in southwestern Uganda and joins few other studies of ambulatory CO monitoring among mixed gender populations in sub-Saharan Africa. The only other study of ambulatory CO levels in Uganda, in which CO exposure concentrations were compared by sex and age strata, similarly found that CO exposure was higher in women as compared to men [20]. Our results are also consistent with work from rural Malawi, where median CO exposure was similar and the odds of respiratory symptoms were likewise higher among those with higher CO exposure [44]. On the other hand, ambulatory CO levels among a cohort in urban Burkina Faso were about twice as high as those measured in our study [13], which may be related to the largely rural location of the participants in our cohort, though investigators identified a similar relationship between ambulatory CO levels and biomass smoke exposure. Ambulatory CO exposure was slightly higher among a rural Kenyan cohort of predominantly women who cooked mostly with firewood [45], which is likely due to the largely female composition of the Kenyan cohort, as meal preparation in East Africa is almost exclusively a woman’s responsibility. Lastly, while investigators from the AIR study in urban Malawi also measured personal CO levels in a case-control study of hospitalized patients with pneumonia [46], summative CO levels were not presented and authors did not investigate correlates of CO exposure, which limits comparisons with our work.

Although the relationship that we identified between biomass use and CO exposure is not novel in sub-Saharan Africa [47–49], our work contributes to the literature in that we estimated personal rather than household or ambient levels. In similar settings, personal and household air quality have been found to correlate poorly [13], so assigning pollution-related health risks based on household pollutant concentrations may introduce exposure misclassification bias. Furthermore, personal CO exposure has been shown to correlate with personal particulate matter exposure in some [20], though not all [50–52], African populations. Thus, our use of personal CO concentrations may minimize exposure misclassification and provides proof-of-concept that that ambulatory CO measurement could be an effective method by which to identify vulnerable subgroups exposed to high levels of air pollution in a rural, RLS where biomass is the leading source of pollution [53].

This is among the first studies to suggest that the relationship between air pollution and respiratory symptoms may differ by HIV serostatus. Higher CO exposure was associated with increased odds of respiratory symptoms among PLWH but not HIV-uninfected study participants in stratified models. This finding is unlikely to be related solely to demographic-related differences in CO exposure because although PLWH were more likely to self-report home charcoal use and live in urban areas (both of which would increase personal CO exposure), they were also less likely to use biomass-based home lighting fuels or be current smokers (both of which would decrease personal CO exposure). Several potential hypotheses may explain this finding. HIV has been associated with increased respiratory morbidity in U.S. [54], European [55] and African [56, 57] cohorts. Those with HIV are thought to experience a higher burden of chronic respiratory symptoms due to a combination of higher susceptibility to lower respiratory tract infections that may cause post-infectious structural lung abnormalities [5, 58, 59] as well as increased risk of chronic lung disease through virally-mediated effects such as accelerated immune senescence, systemic inflammation and direct pulmonary toxicity [6, 60, 61]. Based upon similarities in their relationships with systemic inflammation [7, 60] and lung disease [57, 62–64], chronic HIV infection and air pollution may act synergistically to worsen respiratory morbidity among co-exposed populations. Indeed, we previously found that systemic inflammation varied according to both biomass use and HIV serostatus in this cohort, and that higher indices of systemic inflammation were associated with worse lung function among both PLWH and HIV-uninfected controls [65]. Alternatively, air pollution exposure may increase the risk of pulmonary tuberculosis through smoke-induced endothelial and alveolar damage that facilitates mycobacterial infection [8, 66] and/or altered antimycobacterial innate immunity [67]. Thus, air pollution exposure may potentiate baseline tuberculosis risk among PLWH [68], which may underlie the increased respiratory symptom burden among PLWH as compared to HIV-uninfected participants exposed to air pollution. We did not test for tuberculosis in the cohort, so we could not evaluate the potentially mediating effect of pulmonary tuberculosis on the relationship between CO exposure and respiratory symptoms. If corroborated, the results of our work suggest that the convergence of the HIV and air pollution epidemics on the African continent may underlie the disproportionate regional burden of respiratory diseases.

Our work also furthers the understanding of sex-based respiratory symptom burden in sub-Saharan Africa. Respiratory symptoms are thought to be more prevalent among women in RLS because women are generally responsible for meal preparation and thus exposed to more indoor air pollution. The results of this work may challenge that assumption because the relationship between female sex and respiratory symptoms persisted even after adjusting for personal CO exposure. One potential mechanism that may explain this finding is sex-based differences in susceptibility to the respiratory effects of inhaled pollutants. Tobacco smoke, for example, has a greater impact on lung function among women as compared to men [9, 69], possibly related to sex hormone-based effect potentiation [70, 71]. In a cohort study of 39 healthy young adults, short-term wood smoke particle exposure also differentially impacted influenza virus-associated changes in nasal inflammatory gene expression profiles in a sex-dependent manner [10]. Among those exposed to wood smoke particles before inoculation, virus-associated gene expression was increased among men but decreased among women, which suggests that inhaled pollutants also impact respiratory virus susceptibility differentially based on sex. Consistent with these sex-based differences, we found that at similar levels of exposure, CO-exposed women had increased odds of respiratory symptoms as compared to CO-exposed men, and our group has previously shown that systemic inflammation is higher among women as compared to men in this cohort [26]. Our findings suggest that sex-based differences in biomass exposure levels may only partially explain the association between female sex and respiratory morbidity in RLS.

The main strength of our analysis is the use of a well-characterized, population-based cohort in rural Uganda with repeated measures of air quality. Our use of ambulatory monitors also makes our results less susceptible to exposure misclassification bias compared to area monitors. Our study also has several limitations. Firstly, our study participants did not complete time-activity analyses, so we could not correlate peaks of CO exposure with specific activities, which may have limited our identification of additional predictors of CO exposure. Additionally, we did not formally monitor device use compliance, so some participants may not have worn the devices for the entire sampling period. However, we have no reason to believe that non-use would be non-random, so would suggest that the exposure misclassification bias this may have introduced would be most expected to bias our results towards the null, further underscoring the significance of our findings. Secondly, while repeated CO measurement periods are more likely to approximate chronic exposure compared with single measurements, they may still not be fully reflective of chronic exposures and thus may misclassify exposure. We also use ambulatory CO measures to estimate personal air quality, which is supported by the work of some [17, 18] but not all [50–52] groups who have evaluated the correlation between personal CO and particulate matter exposure in similar settings. Additionally, sputum samples were not available for analysis, so we were unable to evaluate participants for active tuberculosis. However, the degree of viral control and the TB incidence in Uganda make undiagnosed active tuberculosis less likely [72, 73]. Though we identified differences in the relationship between CO exposure and respiratory symptoms in analyses stratified by sex and HIV serostatus, the associated tests for interaction were not statistically significant, so further work is necessary to explore whether sex or HIV serostatus modify the relationship between air pollution and respiratory morbidity. Lastly, while our results are generalizable to adults living in rural Uganda, future studies are needed to evaluate the generalizability of these findings to other priority populations in sub-Saharan Africa, including children.

## Conclusions

In summary, our data offer proof-of-concept that ambulatory CO monitoring is a low-cost and feasible means of assessing personal air quality in a rural sub-Saharan African region, identify biomass smoke as an important source of ambulatory CO exposure, and explore potential differences in pollution-associated respiratory morbidity based on HIV serostatus and sex. These findings are important because they extend our knowledge of which populations are most susceptible to pollution-related respiratory morbidity in resource-limited settings, which is critical to informing policy interventions focused on decreasing respiratory morbidity among the most vulnerable populations. Future work is needed to formally evaluate the potentially interactive relationships between air pollution and both sex and HIV serostatus. If corroborated, the results of our work suggest that the convergence of the HIV and air pollution epidemics may underlie the disproportionate regional burden of respiratory morbidity on the African continent and may highlight vulnerable populations on which future exposure mitigation efforts must focus.

## Additional file

Additional file 1: **Figure S1.** Study Questionnaire. **Table S1.** Cohort Characteristics at baseline comparing participants who completed at least one CO measurement to participants who declined CO measurements. **Table S2.** Correlates of 8-h time weighted average CO exposure > 9 ppm. **Table S3.** Correlates of self-reported respiratory symptoms. **Table S4.** Correlates of self-reported respiratory symptoms, stratified by sex. **Table S5.** Correlates of self-reported respiratory symptoms, stratified by HIV serostatus. **Table S6.** Correlates of self-reported respiratory symptoms, sensitivity analysis replacing the CO exposure variable with the biomass cooking variable (*n* = 415). **Table S7.** Correlates of self-reported respiratory symptoms, sensitivity analysis removing those living in homes where charcoal is used for cooking (*n* = 355). (DOCX 54 kb)

## Data Availability

The datasets used and/or analyzed during the current study are available from the corresponding author on reasonable request.

## Abbreviations

AOR: Adjusted odds ratio
CI: Confidence interval
CO: Carbon monoxide
HIV: Human immunodeficiency virus
IQR: Interquartile range
PLWH: People living with HIV
ppm: Parts per million
RLS: Resource-limited settings
TWA: Time-weighted average
WHO: World health organization
