# Supplementary material for: Personal carbon monoxide exposure, respiratory symptoms, and the potentially modifying roles of sex and HIV infection in rural Uganda: a cohort study
Source: Environ Health. 2019 Aug 20;18:73. doi: 10.1186/s12940-019-0517-z (PMC6701123; doi:10.1186/s12940-019-0517-z)
Supplement: Supplementary file 1 — Figure S1. Study Questionnaire. Table S1. Cohort Characteristics at baseline comparing participants who completed at least one CO measurement to participants who declined CO measurements. Table S2. Correlates of 8-h time weighted average CO exposure > 9 ppm. Table S3. Correlates of self-reported respiratory symptoms. Table S4. Correlates of self-reported respiratory symptoms, stratified by sex. Table S5. Correlates of self-reported respiratory symptoms, stratified by HIV serostatus. Table S6. Correlates of self-reported respiratory symptoms, sensitivity analysis replacing the CO exposure variable with the biomass cooking variable (n = 415). Table S7. Correlates of self-reported respiratory symptoms, sensitivity analysis removing those living in homes where charcoal is used for cooking (n = 355). (DOCX 54 kb) [file 12940_2019_517_MOESM1_ESM.docx]

Data Supplement

**Title:** Personal carbon monoxide exposure, respiratory symptoms, and the potentially modifying roles of sex and HIV infection in rural Uganda: a cohort study

**Authors:** Crystal M. North^1-3^, Piers MacNaughton^2^, Peggy S. Lai^1-3^, Jose Vallarino^2^, Samson Okello^2,4,5^, Bernard Kakuhikire^4^, Alexander C. Tsai^1,3,4^, Marcia C. Castro^2^, Mark J. Siedner^1,3,4^, Joseph G. Allen^2,3^, David C. Christiani^1-3^

**Affiliations:**

^1^Massachusetts General Hospital, Boston, MA; ^2^Harvard T.H. Chan School of Public Health, Boston, MA; ^3^Harvard Medical School, Boston, MA; ^4^Mbarara University of Science and Technology, Mbarara, Uganda; ^5^University of Virginia Health System, Charlottesville, USA

**Figure S1.** Study Questionnaire

Do you currently smoke tobacco, such as cigarettes or pipes?

☐ Yes, I smoke every day

☐ Yes, I smoke but not every day

☐ I do not smoke at all

In the past, have you smoked tobacco such as cigarettes or pipes on a daily basis, less than daily, or not at all?

☐ Yes, I used to smoke every day

☐ Yes, I used to smoke but not every day

☐ No, I have never smoked in my life

What year (or age) did you first begin smoking? _______ YEAR / YEARS OLD (Circle one)

What year (or age) did you quit smoking? ______ YEAR / YEARS OLD (Circle one)

What type of cooking fuel is used at your home on most days?

a. Charcoal ☐ Yes ☐ No

b. Firewood ☐ Yes ☐ No

c. Electricity ☐ Yes ☐ No

e. Kerosene stove ☐ Yes ☐ No

f. Biogas stove ☐ Yes ☐ No

g. Other (specify): _____________________

Where is the cooking done?

☐ a. Inside the main house

☐ b. Inside a building, but in a separate building than the main house

☐ c. Outside

How far away from the main house is the cooking done? ______ (meters)

Are you responsible for meal preparation for your family? ☐ Yes ☐ No

As of last night, how many people spent the night in your home? ______ (people)

Apart from the fuel you mentioned in Question 1 above, is there any other cooking fuel used at your home to make smaller meals? ☐ Yes ☐ No

What type of other cooking fuel is used to make smaller meals at your home?

a. Charcoal ☐ Yes ☐ No

b. Firewood ☐ Yes ☐ No

c. Electricity ☐ Yes ☐ No

e. Kerosene stove ☐ Yes ☐ No

f. Biogas stove ☐ Yes ☐ No

g. Other (specify) ____________________

On average, how many hours do you spend in your house each day? ______ (hours)

How many rooms are in your house? ______ (rooms)

How many windows (or openings in the wall) are in your house? ______ (windows)

How many doors (or openings through which you walk) are in your house? ______ (doors)

Do you burn the trash/rubbish/garbage from your home? ☐ Yes ☐ No

How far away from the house is trash burned? ______ (meters)

How many times each week do you burn your trash/rubbish/garbage?

☐ a. <1 time each week
☐ b. 1-6 times each week

☐ c. At least once per day

What do you use to light your home on most days?

☐ a. None

☐ b. Electricity

☐ c. Solar powered light

☐ d. Kerosene Lamp

☐ e. Candles

☐ f. Batteries

☐ g. Other (specify): ­­­­­_________

How would you rate the overall air quality in your house?

☐ a. Excellent

☐ b. Good

☐ c. Fair

☐ d. Poor

☐ e. Very Poor

Has a physician, other health care worker, or traditional healer ever told you that you have any of the following conditions:

COPD (Lung disease caused by smoking tobacco products or inhaling smoke from charcoal or firewood stoves)?

- 1. ☐ Yes ☐ No
  2. What year (or age) were you diagnosed with COPD? ______ year / years old (circle one)
  3. Have you ever been prescribed medicines for COPD? ☐ Yes ☐ No
  4. Are you currently taking medicines for COPD? ☐ Yes ☐ No

Asthma?

1. ☐ Yes ☐ No
2. What year (or age) were you diagnosed with asthma? ______ year / years old (circle one)
3. At what age was your first attack? ______ (years)
4. Have you ever been prescribed medicines for Asthma? ☐ Yes ☐ No
5. Are you currently taking medicines for Asthma? ☐ Yes ☐ No

Pneumonia?

1. ☐ Yes ☐ No
2. Did you take antibiotics? ☐ Yes ☐ No
3. Were you treated in the hospital? ☐ Yes ☐ No

Tuberculosis?

1. ☐ Yes ☐ No
2. Did you take antibiotics? ☐ Yes ☐ No
3. Were you treated in the hospital? ☐ Yes ☐ No

SYMPTOMS

Do you usually have a cough? ☐ Yes ☐ No

Do you usually cough as much as 4 to 6 times a day and 4 or more days out of the week? ☐ Yes ☐ No

Do you usually cough like this on most days for 3 consecutive months or more during the year? ☐Yes ☐ No

For how many years have you had this cough? _____ (write 0 if less than 1 year)

Do you usually bring up phlegm from your chest? ☐ Yes ☐ No

*(Count phlegm with the first smoke or on first going out-of-doors. Exclude phlegm from the nose. Include swallowed phlegm.)*

Do you usually bring up phlegm like this as much as twice a day and 4 or more days out of the week? ☐ Yes ☐ No

Do you bring up phlegm like this on most days for 3 consecutive months or more during the year? ☐ Yes ☐ No

For how many years have you had trouble with phelgm? ______ (write 0 if less than 1 year)

Does your chest ever sound wheezy or whistling:

When you have a cold? ☐ Yes ☐ No If yes, for how many years? ____ (years)**

Occasionally apart from colds? ☐ Yes ☐ No If yes, for how many years? ____ (years)**

Most days or nights? ☐ Yes ☐ No If yes, for how many years? ____ (years)**

**Write 0 if less than 1 year

Have you ever had an attack of wheezing that made you feel short of breath? ☐Yes ☐ No

Have you ever had 2 or more of these episodes? ☐ Yes ☐ No

Have you ever required medicine or treatment for these attacks? ☐ Yes ☐ No

Are you troubled by shortness of breath when hurrying on flat ground or walking up a slight hill? ☐ Yes ☐ No

Do you have to walk slower than people of your age on the level because of breathlessness?

☐ Yes ☐ No

Do you ever have to stop for breath when walking at your own pace on the level?

☐ Yes ☐ No

Do you ever have to stop for breath after walking about 100 meters or after a few minutes on flat ground? ☐ Yes ☐ No

Are you too breathless to leave the house or breathless on dressing or undressing?

☐ Yes ☐ No

If you get a cold, does it usually (greater than 50% of the time) go to your chest? ☐Yes ☐ No

During the past 3 years, have you had any chest illness that stopped you from working, or kept you indoors at home, or in bed? ☐ Yes ☐ No

Please check the box in the appropriate column if any of your family members have or have ever had in the past the listed conditions.

| **Disease / Condition** | **a. Mother** | **b. Father** | **c. Brother or Sister** |
| --- | --- | --- | --- |
| COPD | ☐ Yes  ☐ No  ☐ Don’t Know | ☐ Yes  ☐ No  ☐ Don’t Know | ☐ Yes  ☐ No  ☐ Don’t Know |
| Asthma | ☐ Yes  ☐ No  ☐ Don’t Know | ☐ Yes  ☐ No  ☐ Don’t Know | ☐ Yes  ☐ No  ☐ Don’t Know |
| Lung Cancer | ☐ Yes  ☐ No  ☐ Don’t Know | ☐ Yes  ☐ No  ☐ Don’t Know | ☐ Yes  ☐ No  ☐ Don’t Know |
| Tuberculosis | ☐ Yes  ☐ No  ☐ Don’t Know | ☐ Yes  ☐ No  ☐ Don’t Know | ☐ Yes  ☐ No  ☐ Don’t Know |
| Pneumonia | ☐ Yes  ☐ No  ☐ Don’t Know | ☐ Yes  ☐ No  ☐ Don’t Know | ☐ Yes  ☐ No  ☐ Don’t Know |

| **Table S1.** Cohort Characteristics at baseline comparing participants who completed at least one CO measurement to participants who declined CO measurements | | | |
| --- | --- | --- | --- |
|  | | | |
| **Characteristics** | **No CO measurements**  **(n = 28)** | **At least 1 CO measurement**  **(n = 260)** | ***p* value** |
| Age, years | 52 [50, 54] | 51 [48, 56] | 0.80 |
| Female sex | 12 (43) | 123 (47) | 0.65 |
| HIV positive | 17 (61) | 131 (50) | 0.30 |
| Smoking History |  |  | 0.89 |
| Never Smoker | 16 (57) | 133 (51) |  |
| Former Smoker | 8 (29) | 86 (33) |  |
| Current Smoker | 4 (14) | 41 (16) |  |
| Farmer | 15 (54) | 177 (68) | 0.12 |
| Rural dwelling | 25 (89) | 221 (85) | 0.78 |
| Education |  |  | 0.04 |
| Did not complete primary school | 19 (68) | 143 (55) |  |
| Completed primary school | 4 (14) | 91 (35) |  |
| Completed secondary school | 5 (18) | 26 (10) |  |
| Self-reported home air quality |  |  | 0.46 |
| Very good/Excellent | 12 (43) | 143 (55) |  |
| Fair | 9 (32) | 69 (27) |  |
| Poor/Very Poor | 7 (25) | 48 (18) |  |
| Cooking fuel at home |  |  | 1.00 |
| Firewood | 24 (86) | 221 (86) |  |
| Charcoal | 4 (14) | 37 (14) |  |
| Cooking Location |  |  | 0.07 |
| Inside the main house | 3 (11) | 6 (2) |  |
| Inside a separate structure | 23 (82) | 232 (89) |  |
| Outside | 2 (7) | 22 (8) |  |
| Distance from house, meters^*^ | 10 [4, 11.5] | 5 [5, 10] | 0.72 |
| Trash burning near home |  |  | 0.45 |
| None | 12 (43) | 98 (38) |  |
| < 1 times/week | 9 (32) | 108 (42) |  |
| 1 - 6 times/week | 6 (21) | 52 (20) |  |
| At least daily | 1 (4) | 2 (1) |  |
| Distance from home, meters^†^ | 17.5 [10, 75] | 20 [10, 50] | 0.93 |
| Home lighting source |  |  | 0.66 |
| Electricity | 8 (29) | 84 (32) |  |
| Kerosene | 17 (61) | 141 (54) |  |
| Battery | 2 (7) | 26 (10) |  |
| Candles | 1 (4) | 6 (2) |  |
| Home ventilation |  |  |  |
| Rooms in house | 3 [2, 4.5] | 3 [3, 4] | 0.73 |
| Windows in house | 4 [2.5, 5] | 4 [3, 5] | 0.70 |
| Doors in house | 2 [1.5, 2] | 2 [2, 2] | 0.55 |
| **Table S1 Legend**  Mean (SD), median [IQR], or n (%) unless otherwise indicated  ^a^ Current/former smokers only; ^b^ For those who don’t cook in the main house; ^c^ For those who burn trash near their home | | | |

| **Table S2.** Correlates of 8-hour time weighted average CO exposure > 9 parts per million | | | | | | |
| --- | --- | --- | --- | --- | --- | --- |
|  | | **Unadjusted** | |  | **Adjusted** | |
| **Characteristic** | | **Odds Ratio** | **95% CI** |  | **Odds Ratio** | **95% CI** |
| Biomass cooking - charcoal | 14.8^***^ | | 7.2 – 30.3 |  | 9.2^***^ | 4.0 – 21.5 |
| Biomass lighting | 0.5^*^ | | 0.3 – 0.9 |  | 1.1 | 0.5 – 2.5 |
| Cooking distance from home^†^ | 0.8 | | 0.7 – 0.8 |  |  |  |
| Trash burning at home | 0.7 | | 0.4 – 1.4 |  |  |  |
| Dry Season | 1.0 | | 0.5 – 1.8 |  |  |  |
| Smoking | 1.2 | | 0.5 – 3.2 |  |  |  |
| Urban residence | 4.5^***^ | | 2.2 – 8.9 |  | 1.9 | 0.8 – 4.5 |
| Home ventilation^‡^ | 0.7^***^ | | 0.6 – 0.8 |  | 0.8 | 0.7 – 1.1 |
| Farmer | 0.3^***^ | | 0.2 – 0.6 |  | 0.7 | 0.3 – 1.6 |
| **Table S2 Legend**  CO: carbon monoxide; CI: confidence interval  Reference categories: biomass: firewood; season: rainy; residence: rural  ^*^ p < 0.05; ^**^ p < 0.01; ^***^ p < 0.001  ^†^ per additional meter  ^‡^ per additional window | | | | | | |

| **Table S3.** Correlates of self-reported respiratory symptoms | | | | | |
| --- | --- | --- | --- | --- | --- |
|  | **Unadjusted** | |  | **Adjusted** | |
| **Characteristic** | **Odds Ratio** | **95% CI** |  | **Odds Ratio** | **95% CI** |
| Age, per year | 1.0 | 0.98 – 1.05 |  |  |  |
| Female sex | 2.7^***^ | 1.7 – 4.2 |  | 3.8^***^ | 2.0 – 7.2 |
| HIV serostatus | 1.7^*^ | 1.1 – 2.7 |  | 1.1 | 0.6 – 2.1 |
| 1-hour CO > 9 ppm | 2.0 | 1.0 – 4.1 |  | 1.9 | 0.9 – 4.0 |
| Dry season | 0.8 | 0.5 – 1.4 |  |  |  |
| Smoking Status |  |  |  |  |  |
| Current | 0.8 | 0.4 – 1.7 |  | 1.1 | 0.4 – 3.1 |
| Former | 1.2 | 0.8 – 2.0 |  | 1.1 | 0.6 – 2.0 |
| Asset Index |  |  |  |  |  |
| Poorest | 1.1 | 0.6 – 2.0 |  |  |  |
| Poorer | 0.7 | 0.4 – 1.3 |  |  |  |
| Richer | 0.8 | 0.5 – 1.5 |  |  |  |
| **Table S3 Legend**  CO: carbon monoxide; CI: confidence interval  Reference categories: sex - male; asset index - richest; smoking status - never-smoker; season - rainy  ^*^ *p* < 0.05; ^**^ *p* < 0.01; ^***^ *p* < 0.001 | | | | | |

| **Table S4.** Correlates of self-reported respiratory symptoms, stratified by sex | | | | | |
| --- | --- | --- | --- | --- | --- |
|  | **Men** (n = 220) | |  | **Women** (n = 200) | |
| **Characteristic** | **Adjusted OR** | **95% CI** |  | **Adjusted OR** | **95% CI** |
| HIV serostatus | 4.6^*^ | 1.3 – 16.1 |  | 0.6 | 0.3 – 1.3 |
| 1-hour CO > 9 ppm^a^ | 1.4 | 0.4 – 4.7 |  | 2.4 | 0.9 – 6.5 |
| Smoking status |  |  |  |  |  |
| Current | 1.4 | 0.3 – 6.2 |  | 2.8 | 0.5 – 17.7 |
| Former | 1.4 | 0.5 – 4.2 |  | 0.9 | 0.4 – 1.9 |
| **Table S4 Legend**  OR: odds ratio; CI: confidence interval; HIV: human immunodeficiency virus; CO: carbon monoxide; ppm: parts per million  Reference categories: sex - male; HIV serostatus - negative; smoking - never-smoker; biomass - firewood  ^*^ *p* < 0.05  ^a^ *p* value for CO x sex interaction term *=* 0.84 | | | | | |

| **Table S5.** Correlates of self-reported respiratory symptoms, stratified by HIV serostatus | | | | | |
| --- | --- | --- | --- | --- | --- |
|  | **HIV Positive** (n = 202) | |  | **HIV Negative** (n = 218) | |
| **Characteristic** | **Adjusted OR** | **95% CI** |  | **Adjusted OR** | **95% CI** |
| Female sex | 1.8 | 0.8 – 4.1 |  | 11.1^***^ | 3.3 – 37.1 |
| 1-hour CO > 9 ppm^a^ | 2.2 | 0.9 – 5.2 |  | 0.9 | 0.1 – 10.1 |
| Smoking status |  |  |  |  |  |
| Current | 2.6 | 0.6 – 11.2 |  | 0.9 | 0.1 – 5.1 |
| Former | 0.8 | 0.3 – 1.9 |  | 1.3 | 0.5 – 3.2 |
| **Table S5 Legend**  OR: odds ratio; CI: confidence interval; HIV: human immunodeficiency virus; CO: carbon monoxide; ppm: parts per million  Reference categories: sex - male; HIV serostatus - negative; smoking - never-smoker; biomass - firewood  ^*^ *p* < 0.05  ^a^ *p* value for CO x HIV interaction term = 0.47 | | | | | |

| **Table S6.** Correlates of self-reported respiratory symptoms, sensitivity analysis replacing the CO exposure variable with the biomass cooking variable (n = 415) | | |
| --- | --- | --- |
|  | | |
| **Characteristic** | **Adjusted OR** | **95% CI** |
| Female sex | 4.0^***^ | 2.1 – 7.6 |
| HIV serostatus | 1.2 | 0.7 – 2.3 |
| Biomass – Charcoal | 1.0 | 0.4 – 2.2 |
| Smoking status |  |  |
| Current | 1.2 | 0.4 – 3.4 |
| Former | 1.1 | 0.6 – 2.1 |
| **Table S6 Legend**  OR: odds ratio; CI: confidence interval; HIV: human immunodeficiency virus; CO: carbon monoxide  Reference categories: sex – male; HIV – HIV negative; smoking – never; biomass – firewood  ^*^ p < 0.001 | | |

| **Table S7.** Correlates of self-reported respiratory symptoms, sensitivity analysis removing those living in homes where charcoal is used for cooking (n = 355) | | |
| --- | --- | --- |
|  | | |
| **Characteristic** | **Adjusted OR** | **95% CI** |
| Female sex | 4.0^*^ | 2.0 – 8.1 |
| HIV serostatus | 1.1 | 0.6 – 2.0 |
| 1-hour CO > 35 ppm | 2.4 | 0.7 – 7.8 |
| Smoking status |  |  |
| Current | 1.0 | 0.3 – 3.2 |
| Former | 1.3 | 0.6 – 2.5 |
| **Table S7 Legend**  OR: odds ratio; CI: confidence interval; HIV: human immunodeficiency virus; CO: carbon monoxide; ppm: parts per million  Reference categories: sex – male; HIV – HIV negative; smoking – never; biomass – firewood  ^*^ p < 0.001 | | |
